# Standardized hysteroscopic management of complete septate uterus with duplicated cervices and longitudinal vaginal septum: A single‐center experience

**DOI:** 10.1002/ijgo.71015

**Published:** 2026-04-14

**Authors:** Ursula Catena, Federica Bernardini, Emma Bonetti Palermo, Federica Pozzati, Eleonora La Fera, Francesca Moro, Antonia Carla Testa

**Affiliations:** ^1^ Dipartimento Scienze della Salute della Donna e del Bambino Fondazione Policlinico Universitario Agostino Gemelli, IRCCS Rome Italy; ^2^ Dipartimento Area Della Donna e Materno Infantile S.C. Ostetricia e Ginecologia, ASST Spedali Civili Brescia Brescia Italy; ^3^ UniCamillus‐International Medical University Rome Italy; ^4^ Università Cattolica del Sacro Cuore Rome Italy

**Keywords:** complete septate uterus, duplicated cervices, longitudinal vaginal septum, Müllerian anomalies, ultrasound‐guided hysteroscopy

## Abstract

**Objective:**

To study perioperative and postoperative complications of a standardized ultrasound‐guided hysteroscopic treatment for complete septate uterus with duplicated cervices and non‐obstructive longitudinal vaginal septum, and to evaluate operative time, symptom changes, and reproductive outcomes.

**Methods:**

Between January 2021 and May 2025, patients diagnosed with complete septate uterus, duplicated cervices, and non‐obstructive longitudinal vaginal septum at a tertiary referral center underwent hysteroscopic resection of the uterine and vaginal septum using a 15 Fr bipolar mini‐resectoscope under real‐time ultrasound guidance, with preservation of the duplicated cervices. Primary outcomes were perioperative and postoperative complications. Secondary outcomes included operative time trends over consecutive cases, symptom changes before and after surgery, and reproductive outcomes (clinical pregnancy rate, live birth rate, miscarriage rate, and pregnancy‐related complications).

**Results:**

Twenty‐eight patients were included. Surgical correction was successfully completed in all cases, with no perioperative or postoperative complications. Median operative time was 61.5 min (range 21–94 min) and decreased modestly over consecutive procedures. At follow up, all patients showed a regular triangular uterine cavity on three‐dimensional transvaginal ultrasound. Median follow up was 24 months (range 3–54 months). Symptoms improved in nearly all patients; only one patient (3.5%) reported persistent dyspareunia. Among the 11 patients who attempted conception, the clinical pregnancy rate was 100% (11/11). The live birth rate per pregnancy was 63.6% (7/11), with two pregnancies ongoing at last follow up.

**Conclusion:**

This standardized ultrasound‐guided hysteroscopic approach using a 15 Fr bipolar mini‐resectoscope for the treatment of complete septate uterus with duplicated cervices and non‐obstructive longitudinal vaginal septum appears safe and reproducible, with favorable anatomic, symptom, and reproductive outcomes.

## 1 INTRODUCTION

The presence of complete septate uterus, duplicated cervices, and non‐obstructive longitudinal vaginal septum represents a particularly rare congenital anomaly of the female genital tract,^1^ classified as U2bC2V1 according to the European Society of Human Reproduction and Embryology/European Society of Gynecological Endoscopy (ESHRE/ESGE) 2013 system.^2^, ^3^ The prevalence of isolated uterine septum is estimated at 1%–2% in the general population,^2^, ^4^ but the incidence of the U2bC2V1 phenotype remains unknown because of its extreme rarity and frequent underdiagnosis.

Women may present with dysmenorrhea, hypermenorrhea, dyspareunia, infertility, recurrent pregnancy loss, and adverse obstetrical outcomes, although some remain asymptomatic.^4^, ^5^, ^6^ Diagnostic standards have shifted from combined laparoscopy and hysteroscopy to non‐invasive imaging, including two‐dimensional transvaginal ultrasound (2D TV‐US) and magnetic resonance imaging (MRI), with further improvements provided by three‐dimensional transvaginal ultrasound (3D TV‐US) and 3D sonohysterography.^3^, ^7^, ^8^ However, both MRI and 3D ultrasound may have limited accuracy in distinguishing duplicated cervices from a single cervix with septum.^8^, ^9^, ^10^ Vaginal assessment by examination is not always feasible in certain populations, such as pediatric patients, individuals with an intact hymen or in cases of obstructive anomalies. Alternatives include vaginoscopy and 3D sonovaginocervicography.^9^, ^10^ In practice, a combination of imaging modalities is essential to improve accuracy. However, there is no consensus on the optimal diagnostic approach in complex cases.^3^

Regarding treatment, emerging evidence supports uterine septum resection in improving reproductive outcomes. Some studies demonstrated the detrimental effect of uterine septum on pregnancy rate, live birth rate, spontaneous abortion, and preterm labor.^11^, ^12^

For U2bC2V1, only case reports and small case series have been published, and no standardized surgical protocol has been established.^3^, ^13^ If surgical treatment is decided, the reference standard remains the resection of both uterine and vaginal septa, preserving the duplicated cervices.^14^, ^15^, ^16^, ^17^ Excision of the endomyometrial tissue within the septum has been proposed.^18^

The present study aimed to evaluate the safety and effectiveness of a standardized ultrasound‐guided hysteroscopic approach for the treatment of U2bC2V1 anomaly. In particular, perioperative and postoperative complications associated with the procedure were assessed. In addition, operative time trends across consecutive cases, changes in patient‐reported symptoms following surgery, and reproductive outcomes, including clinical pregnancy rate, live birth rate, miscarriage rate, and obstetric complications, were explored.

## 2 MATERIALS AND METHODS

### 2.1 Study design and population

This retrospective observational cohort study was conducted between January 2021 and May 2025 at a tertiary referral center (Digital Hysteroscopic Clinic, Class Hysteroscopy, Fondazione Policlinico A. Gemelli IRCCS, Rome, Italy).

Inclusion criteria were a diagnosis of U2bC2V1 anomaly by 3D TV‐US and hysteroscopy. Exclusion criteria included the presence of other Müllerian anomalies not consistent with U2bC2V1 (e.g. obstructive vaginal septum, bicorporeal uterus, single cervix with cervical septum), absence of a definitive diagnosis based on combined 3D TV‐US and hysteroscopy, and previous surgical treatment for the anomaly.

### 2.2 Study objectives

The primary aim of the study was to assess perioperative and postoperative complications, classified according to the Clavien‐Dindo classification system.^19^

Secondary aims included: (1) evaluation of operative time trends over consecutive procedures; (2) assessment of changes in symptoms before and after surgery; and (3) evaluation of reproductive outcomes, including clinical pregnancy rate, live birth rate, miscarriage rate, and obstetric complications.

### 2.3 Diagnosis

Diagnosis was established through a one‐stop setting, combining 2D/3D TV‐US and diagnostic hysteroscopy.^20^ MRI or other additional imaging modalities were not performed.

Pelvic assessment started with 2D TV‐US in longitudinal and transverse planes to evaluate uterus and ovaries and exclude associated gynecologic pathology. All patients underwent 3D TV‐US using a Samsung HM70 EVO with volumetric transvaginal probe (5–9 MHz frequency).

Primary 3D acquisition was performed in the sagittal plane to visualize the endocervical canal and endometrial cavity and to assess the external uterine contour. Automatic volume acquisition was conducted using a sweep angle of 90°. If full acquisition was not feasible in sagittal view, a coronal acquisition was performed. The acquired volume was then processed to analyze the coronal plane at the level of the interostial line. In this view, the following measurements were obtained: interostial line, distance from the interostial line to the uterine serosa, septum length (from the interostial line to the apex of the uterine septum).^21^, ^22^ Ultrasound evaluation of the kidneys was also performed.

Diagnostic hysteroscopy was performed with a 5‐mm continuous flow hysteroscope (Bettocchi, Karl Storz, Germany). Saline solution was used for distention of the uterine cavity and intrauterine pressure was controlled using an Endomat® system (Karl Storz, Germany). Patients presented two hemivaginas separated by a non‐obstructive longitudinal vaginal septum. Hysteroscopic criteria for duplicated cervices included two external uterine orifices, an intercervical cleft, and continuity between vaginal and cervical anomaly.^10^ Entering both cervical canals, the uterus presented two hemicavities separated by the presence of a complete uterine septum.

### 2.4 Intervention

All procedures were performed by a single experienced surgeon (UC), using a standardized minimally invasive approach, following an outpatient model of care.^23^ Patients underwent general anesthesia (laryngeal mask) and were discharged 3 h after the procedure. All women received oral progestin therapy (acetate norethisterone 5 mg or desogestrel 75 μg/day) before surgery for at least 1 month, to reduce endometrial thickness and minimize intraoperative bleeding. All procedures started with diagnostic hysteroscopy with a 5‐mm continuous flow hysteroscope (Bettocchi, Karl Storz, Germany). Saline solution was used for distention of the uterine cavity and intrauterine pressure was controlled using Endomat® system (Karl Storz, Germany).

A 15 Fr bipolar mini‐resectoscope (Karl Storz, Germany) was used for the surgical procedure, consisting of the following steps.^14^

Step 1: Incision of the vaginal septum. Using the Collins loop, the vaginal septum was incised from its apex toward the cervical plane, in a caudo‐cranial direction (Figure 1a,b).

**FIGURE 1:**
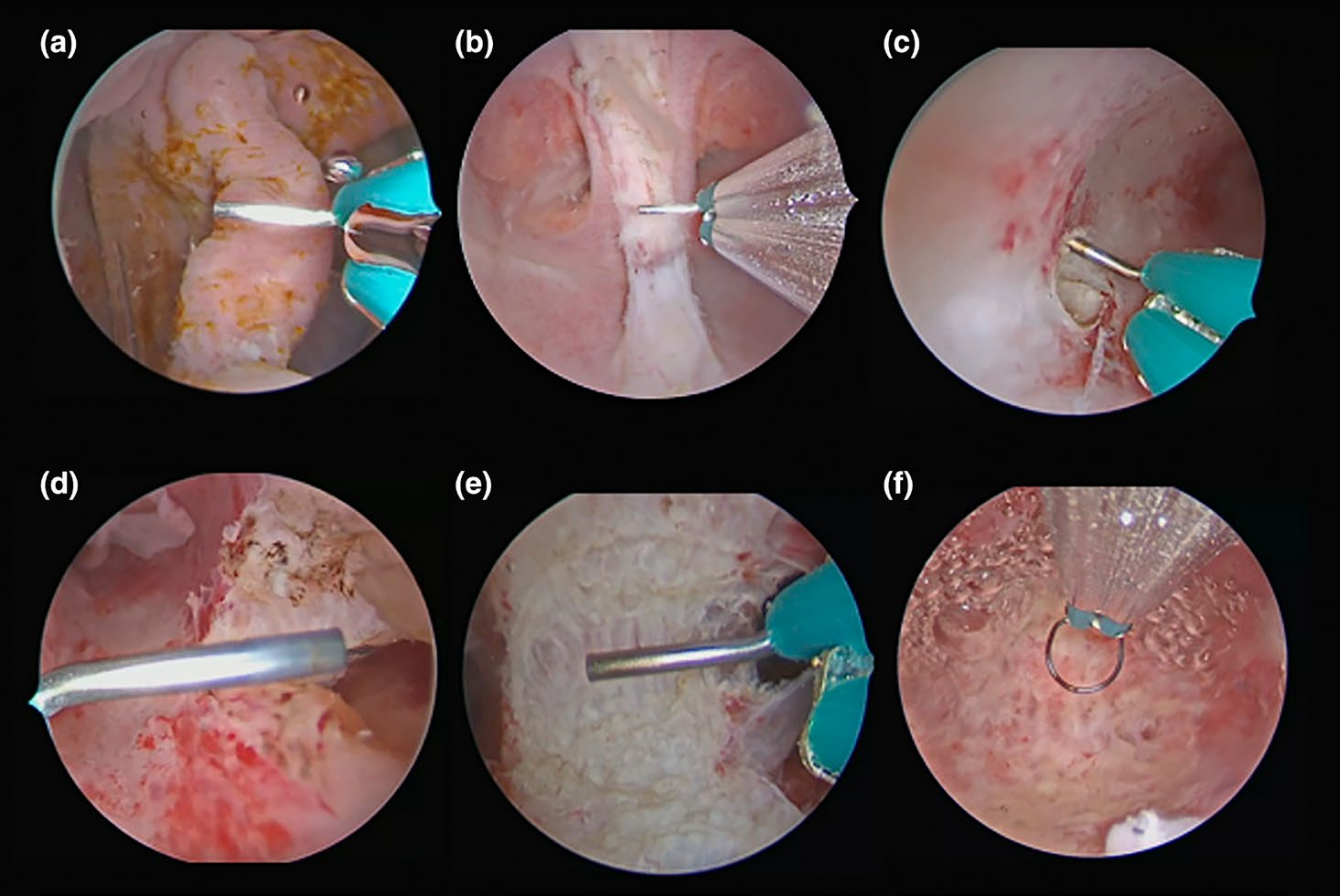
Hysteroscopic standardized surgical steps of the technique to treat U2bC2V1 anomaly (complete septate uterus, duplicated cervices, and non‐obstructive longitudinal vaginal septum) with 15 Fr bipolar mini‐resectoscope. (a) Vaginal septum incision at the caudal part of the vaginal septum with Collins loop. (b) Completion of the vaginal septum incision up to the level of the duplicated cervices. (c) Supracervical incision performed to create communication between the two uterine hemicavities under real‐time transabdominal ultrasound guidance. (d) Creation of communication between the two uterine hemicavities. (e) Incision of the uterine septum in a caudo‐cranial direction toward the interostial plane with Collins loop. (f) Final panoramic hysteroscopic view of the unified uterine cavity, after removal of the redundant endomyometrial tissue with a 90° angled loop.

Step 2: Connection of the two hemicavities. Real‐time 2D ultrasound guidance (transabdominal or transrectal) was used. In coronal view, the isthmic portion of the septum just above the internal uterine orifice was identified (Figure 2a). This region represents the thinnest portion of the septum and serves as the incision site. Maintaining the instrument in one cervical canal, the surgeon started the incision at the designated point using the Collins loop (Figure 1c). The side from which the incision begins was at the surgeon's discretion. Cross‐sectional scan was used to delineate the plane between two hemicavities, reduce false passage risk, and guide latero‐lateral incision until the contralateral hemicavity was entered.

**FIGURE 2:**
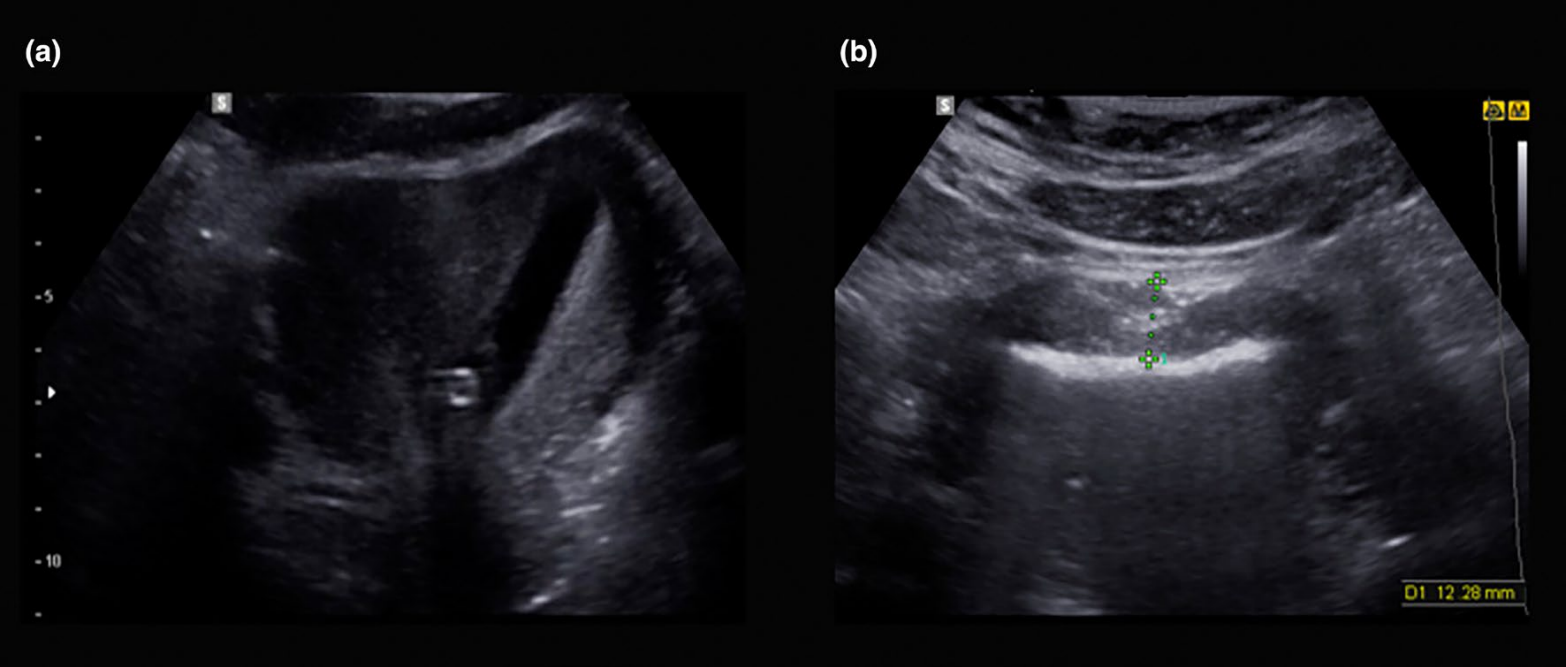
Real‐time transabdominal ultrasound guidance during the standardized surgical technique. (a) Coronal view of the uterus, guiding the supracervical incision between the two uterine hemicavities. (b) Ultrasound measurement of fundal myometrial thickness at the end of the procedure on a coronal view of the uterus.

Step 3: Uterine septum incision. After creating communication (Figure 1d), the septum was incised caudo‐cranially to the interostial plane (Figure 1e). The incision was kept central, using tubal ostia as landmarks. At the end of the procedure, total fundal myometrial thickness was measured under coronal ultrasound guidance (Figure 2b).

Step 4: Resection of redundant endomyometrial tissue. A 90° angled cutting loop was used to resect redundant endomyometrial tissue from anterior and posterior uterine walls (Figure 1f).^18^

### 2.5 Follow up

All patients underwent office hysteroscopy 30–40 days after surgery, with concomitant 2D/3D TV‐US. This procedure was performed in an outpatient setting with the patient awake, with a 5‐mm continuous flow hysteroscope (Bettocchi, Karl Storz, Germany) with saline solution for distention and intrauterine pressure controlled using Endomat® system (Karl Storz, Germany). Cold fundal cuts with 5 Fr scissors were eventually performed to refine the uterine fundus.^24^ All patients underwent 3D TV‐US 1 or 2 months after the office hysteroscopic control, during the secretory phase, to confirm the success of the intervention.

### 2.6 Reproductive outcomes

Reproductive outcomes included clinical pregnancy rate, live birth rate, and miscarriage rate. Clinical pregnancy rate was defined as any pregnancy confirmed by ultrasound per woman. Live birth rate was defined as delivery of a live infant after 24 completed weeks of gestation, calculated per pregnancy. Miscarriage rate was defined as spontaneous loss of a clinical pregnancy before 24 completed weeks, calculated per pregnancy.

### 2.7 Statistical analysis

Analyses were descriptive. Quantitative variables are reported as median and range, and categorical variables as absolute counts and percentages. Peri‐ and postoperative complications (minor and major) were investigated.^19^ Changes in operative time over the years were evaluated using linear regression analysis, assessing the relationship between case number and procedure duration. Pain‐related symptoms (dysmenorrhea and dyspareunia) were assessed using the Numeric Rating Scale (NRS, 0–10) and considered clinically relevant when NRS score was 5 or more.^25^ Differences in symptoms before and after the procedure were described. Reproductive outcomes were reported descriptively (clinical pregnancy rate, live birth rate, miscarriage rate, and pregnancy‐related complications).

### 2.8 Ethical approval

The study adhered to the ethical principles outlined in the Declaration of Helsinki. The study was approved by the regional Medical Ethics Review Committee (Comitato Etico Territoriale Lazio Area 3) under reference number ID 6716 and registered on ClinicalTrials.gov (ID NCT06610864). Written informed consent was obtained from all participants.

## 3 RESULTS

### 3.1 Baseline patient characteristics

A total of 28 consecutive patients with U2bC2V1 anomaly diagnosed and treated at our center during the study period were included, representing all eligible cases. Median age at diagnosis was 30 years (range 18–39 years). Nine patients (32.1%) were asymptomatic. Dysmenorrhea alone was reported by 3 (10.7%), dyspareunia alone by 7 (25.0%), and both symptoms by 11 (39.3%). Two patients (7.2%) had primary infertility. Three patients (10.7%) had experienced a previous miscarriage and 2 (7.1%) a previous elective termination. Two patients (7.1%) had previous pregnancies resulting in preterm cesarean deliveries; in particular, one had two previous preterm cesareans. Baseline characteristics are summarized in Table 1.

**TABLE 1:** Baseline patient characteristics (28 patients).^a^

Clinical features	Value
Age at diagnosis, years	30 (18–39)
Symptoms	
Asymptomatic	9 (32.1%)
Dysmenorrhea^b^	3 (10.7%)
Dyspareunia^b^	3 (10.7%)
Dysmenorrhea + dyspareunia^b^	11 (39.3%)
Infertility	2 (7.2%)
Previous pregnancies	5 (17.9%)
Previous miscarriages	3 (10.7%)
Previous preterm births	2 (7.1%)

^a^ Data are presented as median (range) or number (percentage).

^b^ Symptoms were assessed using the Numeric Rating Scale (0–10) and considered present if the score was 5 or higher.

### 3.2 Diagnosis

In all patients, definitive diagnosis was achieved by combined 3D TV‐US and diagnostic hysteroscopy during a single one‐stop evaluation.^20^ No additional procedures (MRI or diagnostic laparoscopy) were required. No extragenital anomalies, including renal anomalies, were identified in the study population.

### 3.3 Surgical features

Surgery was completed in all patients with complete removal of both vaginal and uterine septa and no peri‐ or postoperative complications. Surgical features are reported in Table 2. Median operative time was 61.5 min (range 21–94 min). Linear regression showed a negative slope (*β* = −0.63; *R* ^2^ = 0.13; *P* = 0.07) between case number and procedure duration, indicating a reduction in procedure time over successive cases (Figure 3).

**TABLE 2:** Surgical features of the population (28 patients).^a^

Surgical features	Value
Surgical time, min	61.5 (21–94)
Presence of communication between the two hemicavities before surgery	
Yes	6 (21.4%)
No	22 (78.6%)
Type of surgery	
One step	25 (89.3%)
Two steps	3 (10.7%)
Myometrial thickness of the uterine fundus at the end of the procedure, mm	10.4 (8–14)
Complications	0 (0.0%)
Fundal incision at hysteroscopic office control	
Yes	21 (75.0%)
No	7 (25.0%)
Myometrial thickness of the uterine fundus at hysteroscopic control 30–40 days later, mm	9.6 (9–13)

^a^ Data are presented as median (range) or number (percentage).

**FIGURE 3:**
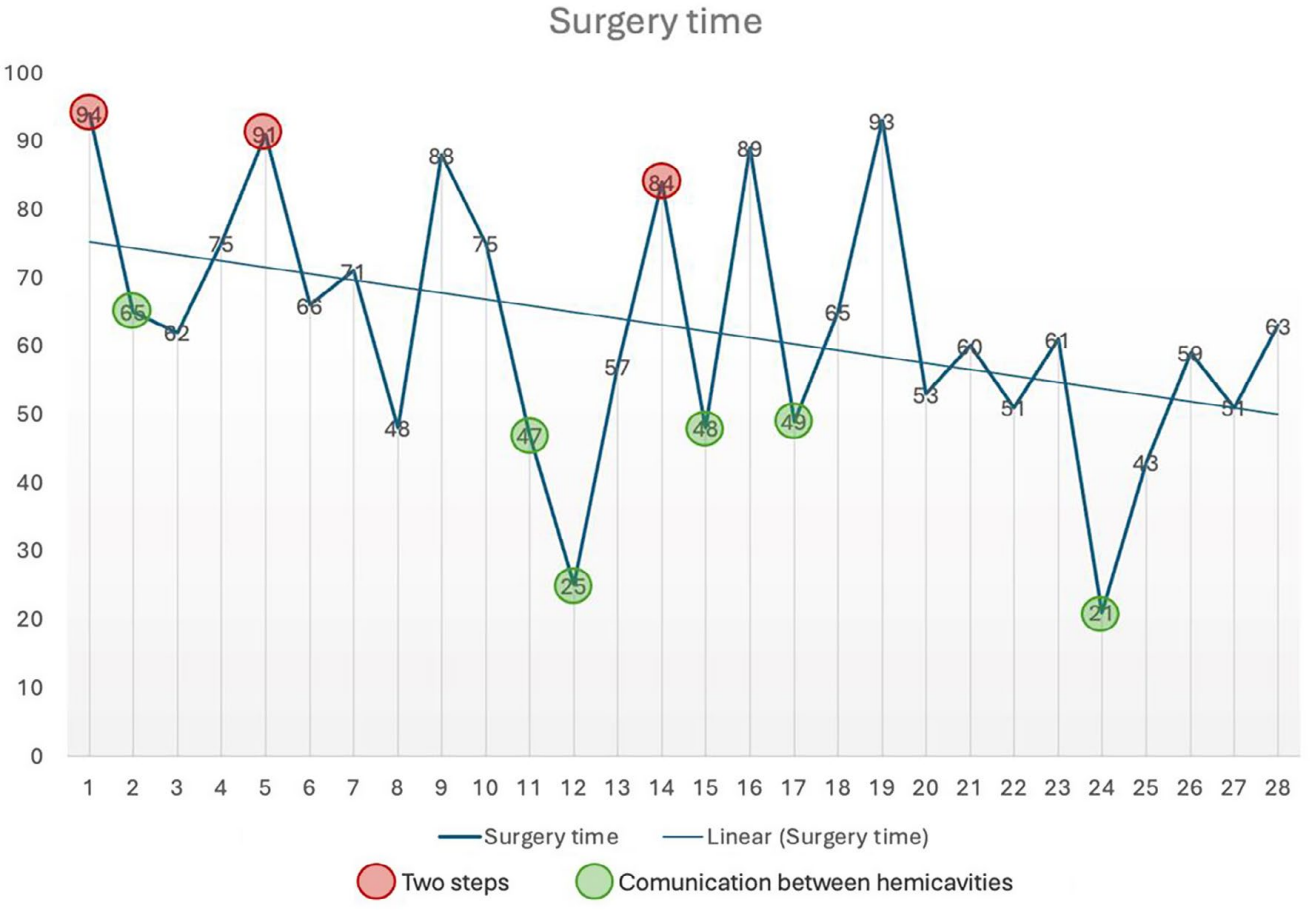
Surgical time of the procedures.

In 22 patients (78.6%), the uterine anomaly was in continuity with the cervical defect, with no communication between the two hemicavities. In 6 cases (21.4%), the two hemicavities were already in communication with a complete uterine septum reaching the internal uterine orifice, not in continuity with the cervical defect. In these cases, step 2 (connection of the two hemicavities) was unnecessary. In one of these cases, previous cesarean delivery precluded distinguishing congenital from acquired communication; in the other four cases, the communication was congenital.

In 25 cases (89.3%), correction was completed in one single surgical step. Vaginal septum was always treated during the first procedure. Three patients (10.7%) required a second surgical step to complete uterine correction. In two cases, step 2 was prolonged with increasing risk of fluid overload, leading to the decision to postpone completion of the procedure to a second surgical step. Notably, the first of these cases was also the first procedure of the cohort. In the second case, severe uterine retroversion limited transabdominal visualization, prolonging operative time. In the remaining case, follow‐up hysteroscopy at 40 days showed residual partial uterine septum requiring reintervention.

Median fundal myometrial thickness at the end of surgery (3D TV‐US) was 10.4 mm (range 8–14 mm). During office hysteroscopic control 30–40 days after surgery, 21 patients (75.0%) underwent a fundal refinement with 5 Fr instruments, optimizing the surgical result. At the 3D TV‐US performed after the fundal incision, all patients presented regular triangular uterine cavity with normal volume; median fundal myometrial thickness was 9.6 mm (range 9–13 mm). Postoperatively, 27 patients (96.4%) were asymptomatic, but one (3.5%) reported persistent dyspareunia. None reported dysmenorrhea.

### 3.4 Reproductive outcomes

Reproductive outcomes are reported in Table 3. Median follow up was 24 months (range 3–54 months). Eleven patients (39.3%, 11/28) attempted conception postoperatively and were included in the analysis of reproductive outcomes. Clinical pregnancy rate was 100% (11/11). At last follow up, seven pregnancies had resulted in full‐term deliveries, corresponding to a live birth rate per pregnancy of 63.6% (7/11). Four pregnancies ended in miscarriage, yielding a miscarriage rate per pregnancy of 36.3% (4/11); one patient experienced two consecutive miscarriages. Two pregnancies were ongoing. One of these was complicated by preterm prelabor rupture of membranes (PPROM) at 22 weeks of pregnancy requiring hospitalization. Among the seven term deliveries, six occurred by cesarean and one by vaginal delivery. No completed pregnancies were complicated by preterm delivery or cervical incompetence.

**TABLE 3:** Reproductive outcomes after treatment.^a^

Outcome	Value
Desire for pregnancy	11/28 (39.3%)
Infertility	1/11 (9.1%)
Assisted reproductive technology	1/11 (9.1%)
Miscarriages	4/11 (36.4%)^b^
Failed transfers	0/11
Preterm pregnancy	0/11
Full term pregnancy	7/11 (63.6%)
Ongoing pregnancy	2/11 (18.2%)

^a^ Data are presented as number (percentage).

^b^ One patient had two miscarriages.

## 4 DISCUSSION

In this cohort, hysteroscopic resection of vaginal and uterine septa for complete septate uterus with duplicated cervices and non‐obstructive longitudinal vaginal septum was completed in all patients without peri‐ or postoperative complications. Operative time decreased over successive cases, symptoms resolved in almost all patients, and live birth rate per pregnancy was favorable.

To our knowledge, this is the largest published cohort of U2bC2V1 patients treated with a standardized technique using a 15 Fr bipolar mini‐resectoscope under ultrasound guidance. Limitations include retrospective design, single‐center setting, small sample size, and reproductive outcomes assessed in a subset of patients, with descriptive analysis only; however, given the rarity of this anomaly, enrollment at a single center remains clinically relevant. Although follow up after metroplasty was limited, the number of pregnancies observed was substantial.

Our findings support the combined use of 3D ultrasound and hysteroscopy for accurate diagnosis of this complex malformation, consistent with previous reports and with the ESHRE/ESGE Thessaloniki consensus, which recommends 3D ultrasound as the primary diagnostic tool and reserves MRI for complex cases or inconclusive ultrasound findings.^3^, ^4^

Cervical anomalies remain the main diagnostic challenge.^7^, ^10^ In our practice, 3D ultrasound provides reconstruction of uterine body features (septum and external contour), whereas lower tract assessment relies on careful vaginoscopy, allowing comprehensive evaluation also in virgo intacta patients. A one‐stop approach (3D ultrasound and hysteroscopy) enabled diagnosis while avoiding repeated visits, additional imaging, and delays.

An interesting finding was pre‐existing isthmic communication between hemicavities in 21.4% of patients, consistent with the bidirectional resorption theory described by McBean and Brumstead,^26^ proposing septal resorption starting at the isthmus and progressing cranially and caudally. This feature simplifies surgery by eliminating step 2, typically the most technically challenging phase under ultrasound guidance.

Hysteroscopic metroplasty is the first‐line treatment for uterine septa, but no consensus guidelines define the optimal management of U2bC2V1. Available evidence suggests that vaginal septum resection improves symptoms and uterine septum resection improves reproductive outcomes, whereas there is no indication to treat duplicated cervix.^27^ Reported techniques historically used loops and fine needles.^13^, ^28^ Recent literature supports the safety and efficacy of the 15 Fr bipolar mini‐resectoscope.^4^, ^14^ Our approach avoids cervical dilation and does not require intrauterine devices to guide septal incision, and no peri‐ or postoperative complications occurred. A limitation is the occasional need for a staged procedure when Step 2 is prolonged. In our series, a two‐step surgical approach occurred in cases with retroverted uterus and more difficult ultrasound guidance, predominantly in early cases, consistent with a progressive learning effect. The reduction in operative time across cases supports a learning curve, although the correlation was modest and not statistically significant (Figure 3).

For vaginal septum management, cold scissors are commonly reported in the literature,^29^ but vaginoscopic use of the 15 Fr bipolar mini‐resectoscope is increasingly described.^30^ In our experience, this method is rapid and safe and can be used also in virgo intacta patients or where there are obstructive vaginal anomalies.^31^ We prefer to incise the vaginal septum first to improve visualization of the cervical anatomy and facilitate differential diagnosis between duplicated cervices and a single cervix with cervical septum, which require different surgical management.^10^

In 2021, Di Spiezio Sardo et al.^32^ described a technique involving the insertion of a Hegar dilator into one of the cervical canals, creating a contralateral bulge that acts as a guide for the incision. Based on the same concept, in 2024 Triantafyllidou et al.^33^ described the use of a Foley balloon catheter. In 2023, our group reported the use of ultrasound as a real‐time guidance tool during hysteroscopic surgery for correction of this anomaly.^14^ The present analysis confirms that ultrasound is a non‐invasive, safe, and effective tool providing continuous control throughout the procedure.

Follow‐up duration in our series (median 24 months) was limited but comparable to other studies. Available data in the literature indicate that a uterine septum is associated with reduced pregnancy rates and live birth rates, and evidence supports metroplasty to improve live birth rates and reduce spontaneous abortions.^11^ Consistent with these findings, reproductive outcomes were favorable in our cohort. Completed pregnancies resulted in term delivery without threatened preterm labor or preterm birth, and newborns were healthy without evidence of fetal growth restriction or small‐for‐gestational‐age status. One case of PPROM at 22 weeks or pregnancy due to chorioamnionitis occurred; given the small sample, no inference can be made regarding associations with PROM/PPROM after septum resection.^11^

In our series, for the first time to our knowledge, one patient delivered vaginally after metroplasty despite cervical duplication, without labor dystocia and with favorable neonatal outcome. This single case does not allow conclusions on generalizability or safety; however, in specialized referral centers, vaginal delivery may be considered in carefully selected cases.

Our study has several important clinical implications. First, the standardization in the diagnosis of complex genital tract anomalies allows for correct counseling of patients before surgical treatment. Second, the standardized minimally invasive approach with 15 Fr bipolar mini‐resectoscope under ultrasound guidance appears safe and effective, with promising reproductive outcomes. Larger multicenter prospective studies and randomized trials are needed to confirm effectiveness.

In conclusion, the present study provides novel insights into diagnosis and management of complete septate uterus, duplicated cervices, and non‐obstructive longitudinal vaginal septum. A one‐stop diagnosis combining 3D TV‐US and hysteroscopy prevents unnecessary delays and reduces the need for additional diagnostic procedures such as MRI or diagnostic laparoscopy. The standardized hysteroscopic technique appears safe and reproducible, and is associated with favorable clinical and reproductive outcomes. Further investigations are warranted to validate these findings and refine surgical strategies.

## AUTHOR CONTRIBUTIONS

All authors contributed to the manuscript at all stages including design, planning, data abstraction, and manuscript writing.

## FUNDING INFORMATION

This research received no specific grant from any funding agency in the public, commercial, or not‐for‐profit sectors.

## CONFLICT OF INTEREST STATEMENT

The authors have no conflicts of interest.

## Data Availability

This is not a randomized controlled trial; however, de‐identified data supporting the findings of this study will be made available to qualified researchers upon reasonable request to the corresponding author.
